# Towards a physically more active lifestyle based on one’s own values: the results of a randomized controlled trial among physically inactive adults

**DOI:** 10.1186/s12889-015-1604-x

**Published:** 2015-03-18

**Authors:** Anu Maarit Kangasniemi, Raimo Lappalainen, Anna Kankaanpää, Asko Tolvanen, Tuija Tammelin

**Affiliations:** LIKES─Research Center for Sport and Health Sciences, Viitaniementie 15 a, 40720 Jyväskylä, Finland; Department of Psychology, University of Jyväskylä, Jyväskylä, Finland

**Keywords:** Acceptance and commitment therapy, ACT, Physical activity, Behaviour, Effectiveness, Adults, Psychological well-being

## Abstract

**Background:**

The high prevalence of physical inactivity has led to a search for novel and feasible interventions that will enhance physical activity, especially among the least physically active individuals. This randomized controlled trial aimed to determine the effectiveness of a value-based intervention to promote a physically more active lifestyle among physically inactive adults. The framework of the study was based on Acceptance and Commitment Therapy (ACT).

**Methods:**

Physically inactive participants aged 30 to 50 years (n = 138) were randomly allocated to a feedback (FB, n = 69) or an acceptance- and commitment-based group (ACT + FB, n = 69). Both groups received written feedback about their objectively measured physical activity and were offered a body composition analysis. In addition, the participants in the ACT + FB group attended six group sessions and were given a pedometer for self-monitoring their physical activity during the nine-week intervention. The primary outcome was physical activity. In addition, participants’ cognitions related to exercise and physical activity were evaluated at baseline and at three- and six-month follow-ups. The changes in mean physical activity level were analysed using multilevel random regression and rank order stability, using the structural equation model.

**Results:**

Participants in both groups increased their objectively measured and self-reported physical activity with high individual differences. No difference was observed in the change of physical activity level between the FB and ACT + FB groups over time. However, the cognitions related to physical activity and exercise improved more in the ACT + FB group than in the FB group. In addition, after re-analyzing the data among the non-depressive participants, higher stability was observed in objectively measured physical activity at the individual level between the three- and six-month follow-ups in the ACT + FB group as compared to FB group.

**Conclusions:**

Acceptance- and commitment-based group intervention, combined with the self-monitoring of physical activity, was beneficial in supporting the cognition related to exercise and physical activity, and brought more stability to the individual level physical activity behaviour change, especially among the non-depressive participants.

**Trial registration:**

ClinicalTrials.gov, number NCT01796990. Registered in February 2013.

**Electronic supplementary material:**

The online version of this article (doi:10.1186/s12889-015-1604-x) contains supplementary material, which is available to authorized users.

## Background

As the rates of physical inactivity are increasing worldwide, effective and new methods are urgently needed to enhance a physically more active lifestyle. Physical inactivity causes many health problems and non-communicable diseases (e.g., coronary heart disease, type 2 diabetes, breast and colon cancer) leading to 9% of premature deaths worldwide [1]. These diseases are a great burden on health care systems and economies because of the costs related to the treatment of diseases and absence from work. Physical inactivity is also associated with depression [2-5] and the association seems to be bi-directional. Greater levels of physical activity enhance psychological well-being by reducing the risk of depression [2,3,5]. Depression, in turn, may contribute to a sedentary lifestyle and poor adherence to physical activity behaviour [4,6,7].

Behavioural interventions have been shown to have only a small [8,9] to moderate [10] effect on self-reported physical activity levels. Despite the relatively poor effectiveness, the interventions that are theory-based [11] and include self-regulatory (e.g., self-monitoring, feedback, goal-setting, etc.) constructs appeared to be more effective than other types of interventions [9,12]. Pedometers have also been used combined with other techniques and the results support their efficacy in increasing physical activity [13]. Beside the effective methods to increase physical activity level, interventions that support small changes and long-term maintenance are also warranted. Among physically inactive adults, even small improvements in physical activity can be beneficial for health [14].

Acceptance and Commitment therapy (ACT) is a new behavioural therapy approach that aims to increase psychological flexibility based on one’s own values in life [15]. Psychological flexibility refers to the ability to be in the present moment with full awareness and openness to experiences. The motivation to behaviour change is based on one’s own values and the important things in life. The ACT approach is based on a philosophy of science called functional contextualism. The focus is on the actions or behaviour that work for the person in his own life circumstance [16], instead of concentrating on following a professional’s advices or recommendations (e.g., 10 000 steps a day) as the basis of changing behaviour. In addition, the purpose is to make changes that are individually important as a regular part of the lifestyle. In many forms of problematic behaviour, the flexible processes are often absent [17]. From the ACT perspective this inflexibility or human suffering is the result of two key processes; cognitive fusion and experiential avoidance. Cognitive fusion refers to the tendency in which people strongly believe the literal content of their mind, thoughts or feelings. Experiential avoidance is in turn a consequence of fusing cognitions of the mind that encourage people to suppress, control or eliminate these experiences, which have been evaluated as distressing. From the perspective of enhancing physical activity, fusion with own thinking patterns or explanations together with avoidance of the physical activity situations which are evaluated as aversive, can partly prevent people from making a change and becoming physically active.

The ACT intervention targets to increase more adaptive behavior by decreasing the consequences of cognitive fusion and experiential avoidance. Thus, one central part of the process of change is related to the skills how to deal with the difficult cognitions, thoughts and emotions, and become aware of the automatic behaviour or thinking patterns through mindfulness skills. In mindfulness training, the goal is to maintain awareness moment by moment, disengaging oneself from beliefs, thoughts and emotions in a non-judgmental manner [18,19]. Several studies have shown that mindfulness skills are associated with better well-being [20,21] and are useful in disengaging individuals from their automatic thoughts, habits, and unhealthy behaviour patterns [22].

Previous studies have shown that higher levels of physical activity are associated with greater levels of mindfulness [23-28]. Furthermore, there is some research evidence to show that mindfulness and acceptance based behavioural approaches actually enhance physical activity [29], or combine with weight related goals [29-32]. A pilot study among college students showed that a short ACT based group intervention was superior to an education group in increasing physical activity levels [29]. Another pilot study among adult cardiac outpatients showed that participants of the acceptance based behaviour therapy (ABBT) reported high treatment satisfaction, comprehension and made positive changes in diet and moderate increases in physical activity [30]. Tapper et al. [32] examined the ACT based intervention for weight loss for women. Participants in the ACT based intervention showed significantly greater increases in physical activity compared to controls. However, in ABBT intervention to facilitate weight gain prevention among college students, significant decreases were observed in body weight and body mass index, but not in physical activity compared to the control group at 6 weeks [31]. ACT has also been successfully used in the treatment of several health related problems (e.g., chronic pain [33], type II diabetes [34] and weight regain among bariatric surgery patients [35,36]). Apart from its general effectiveness in affecting change, ACT is also associated with the maintenance of change [37,38]. This study aimed to investigate the effectiveness of the feedback only (FB) versus the combination of acceptance- and commitment-based group intervention, including self-monitoring of physical activity and feedback (ACT + FB) first on physical activity and second on the cognitions related to exercise and physical activity among physically inactive adults. A further aim was to explore the stability of individual changes in physical activity between the groups. We hypothesized that ACT + FB group is more effective in enhancing physical activity and the cognitions related to physical activity than the FB group.

## Methods

### Study design and population

**Figure 1:**
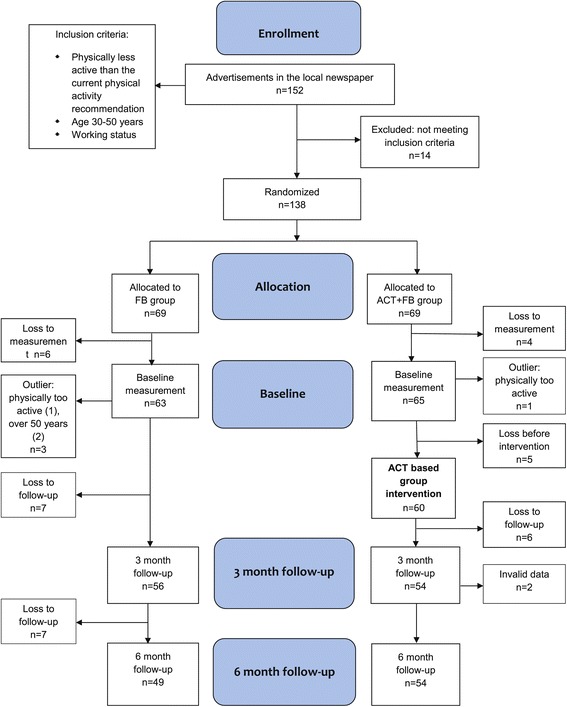
**Flow diagram and the progress of the study.**

The design of the study was a randomized controlled trial (Figure 1) that been described earlier in detail [39]. The data were collected in two phases in order to detect a medium-sized effect at the power level of 85%. According to the power calculations sample size of 100 (50 per a group) would be sufficient to detect a difference in the change of health-enhancing physical activity (HEPA, min/day) between the ACT + FB and FB groups equal to 5 minutes per day. The first phase was carried out during the autumn of 2011 and the second phase during the autumn of 2012. The protocol was approved by the Scientific Ethics Committee of the University of Jyväskylä, Finland. The trial is registered with ClinicalTrials.gov, number NCT01796990. The study population consisted of working adults aged 30 to 50 years who were physically inactive (defined as not meeting the current physical activity recommendations) [40]. Physically inactive adults were recruited by advertisements in the local newspaper. All interested individuals were screened in more detail through an online questionnaire. The criteria for selection were: 1) age 30–50 years, 2) working status, and 3) not meeting the current physical activity recommendations [40]. All eligible participants received written confirmation of their acceptance into the study and were informed about the study protocol. The participants gave written informed consent if they agreed to enroll in the study. The flow chart of the trial is shown in Figure 1. Altogether, 138 participants were randomly assigned to the two parallel study groups, the feedback group, FB (n = 69) and the acceptance- and commitment-based group, including also feedback, ACT + FB (n = 69). At the baseline 128 participants (92.7%) attended the measurements, of whom 124 participants met the criteria and were included in the analysis. After three months, 110 participants (79.7%) completed the measurements. In the second follow-up, after six months, 103 participants (74.6%) completed the measurements. The reasons for drop out were 1) lack of time (n = 7), 2) pregnancy (n = 1), 3) a death of a close relative (n = 1), and 4) no specified reason (n = 26).

### Interventions

#### Feedback group (FB)

The participants in the FB group got written feedback about their objectively measured physical activity level at the baseline and at the three- and six-month follow-ups. In addition, during these measurement periods (seven days), participants were also asked to keep a diary of their physical activities. This individual written feedback included information on participants’ objectively measured daily physical activity level in comparison with the current physical activity recommendations by using histograms. The first histogram described how much time was spent during the last seven days on at least moderate intensity physical activity, lasting at least 10 minutes at a time. In addition, the second histogram described how much total time was spent on physical activity of at least moderate intensity. The feedback also included the amount of daily steps during the week and the time spent on the activities, which were reported in the diary. Individual feedback was sent home in the mail and did not include face-to-face interaction. As an incentive for participation, participants also had an opportunity to attend a body composition analysis and receive a short personal interpretation and feedback of the results at the LIKES Research Center for Sport and Health Sciences.

#### The acceptance and commitment based group (ACT + FB)

ACT + FB group received the same feedback procedures as the FB group. In addition, they participated in the ACT-based group program, which included also self-monitoring of physical activity. The intervention program consisted of six group sessions, each lasting for about 90 minutes, during a nine-week period [39]. Participants got their own workbook, which contained short descriptions of the sessions, as well as space for individual reflections and notes. The program aimed to enhance a physically active lifestyle and well-being through important life values and to build committed action based on the chosen important values (Table 1). The program started with an analysis of health behaviour and reflections about one’s own values and important things in life. After value clarification, participants made concrete action plans and defined their own goals for change. The participants were recommended to choose small and feasible changes related to their everyday life, which can be performed every day (e.g., using the stairs, actively commuting to work), rather than demanding exercise goals. Importance was also placed on self-selected goals and actions that support individually important values. In order to achieve one’s own motivation and find values for the change, the participants in the group were treated individually in an autonomy-supported way. In addition to the special topics of each group session (Table 1), every session included mindfulness exercises, pair and group discussions, and homework related to the session's topic. The aim of the mindfulness exercises was to teach new skills to overcome thoughts related to barriers and the ability to concentrate on the present moment. The program did not include psycho-educational elements or direct health counselling, information about the health benefits of physical activity, or concrete physical exercise. In addition, participants in the ACT + FB group were given a pedometer for self-monitoring daily physical activity during the nine-week intervention.

**Table 1:** **Content of the six group sessions in the acceptance- and commitment-based group**

**Topics of the group sessions**	**Key points and aims of the sessions**
1. Health behaviour	What are the factors affecting my health behaviour and well-being? What is the direction I want to move? What are the steps I have to take in order to try to change my well-being?
2. Values and important things in life	What are the most important values for me? Am I living or behaving according to my values?
3. Value-based actions and barriers	What are my specific goals and actions that support my valued behaviour? What kind of subjective barriers or explanations do I have in relation to my physical activity?
4. Living in the present moment and self-regulation skills	How do I contact the present moment? How do I use mindfulness skills in order to be more aware of my own behaviour in everyday life?
5. Self-processes and physical activity	How do I see myself and how does it affect my behaviour? Can I be more aware of the way I am thinking about myself and learn nonreactive ways to respond to these thoughts?
6. Flexible actions	How am I doing? What are the actions that help me to achieve my desired outcomes? Do I need to change my goals? Am I living according to my values? Can I be more flexible in my behaviour and physically active lifestyle?

### Measurements

Measurements took place at baseline and at three and six months after the baseline (Figure 1). The baseline demographic characteristics of the participants were also recorded through a questionnaire, including socioeconomic, physical, and psychological health variables. The primary measure of outcome was physical activity. The focus was especially on the objectively measured health-enhancing physical activity (HEPA) [39]. In addition, secondary outcomes, cognitions related to physical activity, such as beliefs and intentions for exercise, as well as the acceptance of psychological and physical discomfort related to physical activity were measured with self-administered questionnaires.

### Physical activity behaviour

#### Objectively measured physical activity

Physical activity was measured objectively by using an accelerometer (ActiGraph GT1M, GT3X, Actigraph, Pensacola, Florida). The ActiGraph accelerometer is a small, light instrument that records integrated acceleration information as an activity count, providing an objective estimate of the intensity of vertical bodily movement. Participants were instructed to wear the accelerometer, attached by an elastic belt on the right hip, during all waking hours for seven consecutive days [41]. The outcome variables were time spent on health-enhancing physical activity (HEPA, min/day) and time spent on moderate-to-vigorous intensity physical activity (MVPA time, min/day) [42]. HEPA time was defined as continuous MVPA lasting at least 10 minutes at a time, according to the current physical activity recommendation [40]. The validity and reliability of the Actigraph GTX3 has been shown to be similar to the GT1M devices in laboratory testing and for the measurement of everyday activities [43,44]. The ActiLife accelerometer software (ActiLife version 5; http://support.theactigraph.com/dl/ActiLife-software) was used for data collection. Epoch length used for analysis was 60 seconds, and non-wearing time was identified as a continuous zero registered for >60 minutes. Customised software was used for data reduction and analysis. A cut-off value of 1952 counts per minute (cpm) was used for MVPA [45]. In order to meet at least 80% of the data reliability criterion [41], at least three days of the seven days and for a minimum of 500 minutes per day was set as a minimum criterion for the representative data.

#### Self-reported physical activity

Self-reported physical activity was measured with questions related to participants’ MVPA during the last seven days [30]. Respondents were told to include all activity for which the physical effort was moderate or harder, including transportation to work and leisure-time physical activity. This kind of activity accelerates the heart rate and breathing (e.g., brisk walking, running, and heavy gardening). The physical activity level was determined by the questions: “During the last 7 days, on how many days did you carry out at least moderate intensity physical activity that lasted for at least 10 minutes each time and for a total of at least 30 minutes during one day?” The response options ranged from 0 to 7 days per week. In addition, they were asked “How much time in total did you spend doing this type of physical activity during leisure time?” Estimate was rounded to the nearest half an hour [46].

### Cognitions related to physical activity and exercise

#### Beliefs and intentions for exercise

Intentions and beliefs for physical activity and exercise were measured with the self-administered questionnaires. Adoption self-efficacy concerning exercise was evaluated with five items (α = .84) [47,48], e.g., I can manage to carry out my exercise intentions even if I need a long time to develop the necessary routines, self-efficacy related to barriers regarding exercise with five items (α = .84) [47,49], e.g., I can manage to carry out my exercise intentions even if I have problems and worries, action planning for exercise with four items (α = .92) [48,50], e.g., I have made a detailed plan regarding when to exercise and coping planning for exercise with four items (α = .75) [51,52], e.g., I have made a detailed plan regarding what to do if something interferes with my plans. The response alternatives ranged from 1 (very certain I cannot) to 4 (very certain I can). In the present study, the internal consistency of the questionnaire was good.

#### Acceptance of psychological and physical discomfort related to physical activity

The Physical Activity Acceptance Questionnaire, PA-AAQ, measured the acceptance of psychological and physical discomfort related to physical activity and included 12 items [53] (e.g., I continue to exercise, even when I have the desire to stay home or do something else; I am committing to being physically active no matter what feels uncomfortable or challenging about that). The participants were instructed to answer as follows: “Below you will find the list of statements. Please rate the truth of each statement as it applies to you. Use the following rating scale to make your choices.” The rating scale ranged from 1 (never true) to 7 (always true)”. Higher total scores indicated a greater amount of acceptance of discomfort related to physical activity.

### Depressive symptoms

The Beck Depression Inventory, BDI-II [54,55], was used to measure various characteristics of depression. The BDI-II is a 21-item scale measuring depressive symptoms, including components with cognitive, behavioural, affective and somatic aspects. Based on the scores, depressive symptoms were categorized into two groups: not at all or minimal depression (0–13 points) and at least mild depression (≥14 points), which is used as a cut-off score for the psychiatric screening purposes to detect depressive participants.

### Statistical analysis

Data were analysed using the Mplus statistical package 7.1 (Muthén, L.K & Muthén, B.O, 2001) [56]. First, the intervention effect on objectively measured physical activity, as well as on secondary outcome variables, was examined between baseline (t1) and three-month follow-up (t2) and between t2 and six-month follow-up (t3) using multilevel random regression model. Each outcome variable was regressed on two dummy coded variables c1 (0,1,1 for time t = 1,2,3, respectively) and c2 (0,0,1, for time t = 1,2,3, respectively) in within level. After that, this random regression was regressed on condition in between level. The difference in mean change within and between the groups was tested using Wald test. Further if Wald test was statistically significant, the change from t1 to t2 and t2 to t3 were tested using two dummy coded variables c1 and c2. Cohen’s *d* was used as a measure of effect size for within-group change, and it was calculated by dividing the difference of the means by pooled standard deviation at baseline. The effect size for between group change of 0.2-0.3 is considered small, 0.66 medium, and 0.81 large [57].

After that, differences in the stability of physical activity (PA) between the ACT + FB and FB groups were examined in objectively measured and self-reported physical activity. At first, path model (PA at t2 was predicted by PA at t1 and PA at t3 was predicted by PA at t1 and t2) using structural equation model was fitted in both groups by using the multiple-group analysis method and all the regression coefficients were estimated freely (saturated model). After that, the more constrained model in which the corresponding regression coefficients were fixed to be equal across groups was estimated. The Satorra-Bentler scaled *χ*^2^-difference test was used to compare the nested models (in this case, the *χ*^2−^difference test is equal to the *χ*^2^-test of the constrained model). The estimation results of the saturated model were reported. In addition, the equality of each regression coefficient between groups was tested for significance. In both multilevel and structural equation model full information maximum likelihood (FIML) estimation under the assumption of data missing at random (MAR) was used in analyzing incomplete data. Thus, incomplete data were analysed according to intention to treatment principles. As the normality assumption is violated, the maximum likelihood with robust standard errors (MLR) was used.

## Results

Characteristics of the participants are summarized in the Table 2. The mean age of the participants was 43.5 years and most of them were females (83.3%). The groups were similar in terms of age, gender, marital status, education level, number of children under seven years in the household, body height, depressive symptoms and diagnosed mental and physical conditions. There was no significant difference in the mean score values describing depressive symptoms (BDI-II) between the groups at baseline. However, at least mild depression (BDI-II ≥14) was detected among 25.0% in the ACT + FB group and among 11.7% in the FB group, based on the Beck’s Depression Inventory-II (*p* = 0.056). In addition, the participants in the ACT + FB group had significantly higher body weight compared to the FB group, but no difference was observed in BMI between the groups. Overall, most of the participants were overweight or obese. Background characteristics of the participants with complete data did not differ from those participants who dropped out from the follow-ups, except for one detail: The dropouts reported more children younger than seven years in their household (52.0% among dropouts vs. 15.2% among those with complete data *p* < 0.001). The participants in the ACT + FB groups showed rather good acceptance and adherence to the group intervention. 75% of the participants attended five or all six of the six sessions. The attendance varied from one to all six sessions. A pedometer was used by 56% of the participants in ACT + FB group for at least 90% of the total time during the nine-week intervention.

**Table 2:** **Background characteristic of the feedback, FB and acceptance and commitment based, ACT+FB groups**

**Background variables**	**FB group(n = 60)**		**ACT+FB group (n = 64)**		***p*** **-value** ^**a**^
	***%***	**Mean (SD** ^**b**^ **)**	***%***	**Mean (SD** ^***b***^ **)**	
Age, years		43.0 (5.3)		43.9 (4.8)	0.338
Gender					
Women	85.0		79.7		0.439
Men	15.0		20.3		
Marital status					
In a relationship	73.3		75.0		0.351
Divorced	15.3		20.3		
Single	8.3		4.7		
Widowed	3.3				
Other					
Highest education level					0.677
Vocational school	11.9		10.9		
High school	5.1		7.8		
Polytechnic/Bachelor’s degree	54.2		60.9		
Master’s degree/PhD	28.8		20.3		
Children (<7 years) in the same household	23.3		21.9		0.846
Body height, cm		168.7 (8.0)		170.1 (8.4)	0.365
Body weight, kg		79.2 (13.3)		84.8 (17.2)	0.045
Body mass index		27.9 (4.9)		29.4 (5.7)	0.116
<25 (normal weight)	33.3		24.2		
25-30 (overweight)	38.3		29.0		
>30 (obese)	28.3		46.8		0.110
Diagnosed mental health conditions	18.3		24.1		0.440
Depressive symptoms (BDI-II^c^)		8.0 (6.4)		9.1 (6.5)	0.320
Depressive symptoms (BDI-II ≥14^d^)	11.7		25.0		0.056
Diagnosed physical conditions	36.7		41.4		0.600

^a^Independent samples *t*-test or Pearson’s chi-squared test for group difference.

^b^SD, standard deviation, ^c^BDI-II, Beck’s depression inventory, ^d^BDI-II, cut-off score for at least mild depression.

### Effects and change in the mean levels

#### Physical activity behaviour

The results of the FB group showed statistically significant change over time in objectively measured HEPA (*χ*^2^ = 8.585, *p =* 0*.*014) and self-reported physical activity (*χ*^2^ = 8.755, *p =* 0*.*013). In the FB group the change was observed in HEPA time between baseline and three-month follow-up (t1 vs. t2: *p* = 0.04). However, for self-reported physical activity further analysis did not show statically significant changes between the different time points. In the ACT + FB group significant change over time was observed in objectively measured HEPA (*χ*^2^ = 13.114, *p =* 0*.*001) and self-reported physical activity (*χ*^2^ = 9.606, *p =* 0.008). The change was observed in HEPA between baseline and three-month follow-up (t1 vs. t2: *p* = 0.06) and self-reported measured physical activity between three- and six-month follow-ups (t2 vs. t3: *p* = 0.006). No significant improvements were observed in MVPA in either group (FB: *χ*^2^ = 3.448, *p =* 0*.*178; ACT + FB: *χ*^2^ = 3.699, *p =* 0*.*157). There was no difference between FB and ACT + FB groups in the change of HEPA (*χ*^2^ = 0.557, *p =* 0*.*757), MVPA time (*χ*^2^ = 0.912, *p =* 0*.*634) and self-reported physical activity (*χ*^2^ = 3.734, *p* = 0.155) over time. Both FB and ACT + FB groups improved their objectively measured HEPA time and self-reported physical activity during the follow-up period with high variation (see Table 3 and Additional file 1).

**Table 3:** **Objectively measured physical activity, self-reported physical activity and psychological variables related to physical activity**

	**FB group Mean (SD)**					**ACT+ FB group Mean (SD)**				
	**Baseline t1**	**3 month follow-up t2**	**6 month follow-up t3**	**Cohen** ***d*** **t1 vs. t2**	**Cohen** ***d*** **t1 vs. t3**	**Baseline t1**	**3 month follow-up t2**	**6 month follow-up t3**	**Cohen** ***d*** **t1 vs. t2**	**Cohen** ***d*** **t1 vs. t3**
**Objectively measured PA min/day**										
MVPA	22.8 (12.5)	24.3 (14.9)	26.6 (16.8)	0.12	0.31	26.2 (12.4)	27.4 (14.5)	29.5 (17.6)	0.10	0.27
HEPA	5.8 (6.1)	9.0 (11.8)	10.3 (13.4)	0.47	0.66	6.4 (7.4)	10.1 (9.9)	11.9 (14.0)	0.54	0.81
**Self-reported PA min/day**	11.6 (11.4)	15.6 (15.7)	18.9 (14.5)	0.30	0.56	15.4 (14.6)	17.4 (14.3)	25.1 (18.0)	0.15	0.74
**Adoption self- efficacy**	13.7 (2.5)	13.7 (3)	13.9 (3.7)	0	0.08	14.2 (2.7)	14.7 (2.6)	15.3 (3.1)	0.19	0.42
**Barriers regarding exercise**	11.9 (2.3)	11.7 (2.8)	11.9 (3.1)	−0.08	0	11.4 (2.9)	12.8 (2.6)	13.0 (2.8)	0.53	0.61
**Action planning**	8.8 (3.4)	9.3 (3.7)	9.8 (3.5)	0.16	0.32	8.0 (2.9)	11.2 (3)	11 (2.9)	1.02	0.95
**Coping planning**	6.2 (2.4)	7.2 (2.7)	7.6 (3.4)	0.43	0.61	5.7 (2.2)	9.5 (3)	9.1 (3.1)	1.65	1.48
**PA-AAQ** ^**a**^	41.8 (11.7)	46.5 (13.2)	45.2 (12.7)	0.45	0.32	44.4 (9.2)	52.6 (9.7)	54.5 (9.7)	0.78	0.96

PA, physical activity; MVPA, moderate-to-vigorous intensity physical activity; HEPA, health-enhancing physical activity; PA-AAQ, physical activity acceptance questionnaire.

^a^PA-AAQ was measured only during the second trial (FB group: n = 27, ACT+FB group: n = 32).

#### Cognitions related to exercise and physical activity

There were no change over time in the FB group in self-efficacy related to the adoption of exercise (*χ*^2^ = 0.326, *p =* 0*.*850), self-efficacy when facing the barriers of exercise (*χ*^2^ = 0.749, *p =* 0*.*688), the change of action planning (*χ*^2^ = 2.621, *p =* 0*.*270) as well as in the acceptance of psychological and physical discomfort related to physical activity (*χ*^2^ = 3.739, *p =* 0*.*154). The change in coping planning related to own exercise intentions was significant (*χ*^2^ = 11.913, *p =* 0*.*003) between baseline and three-month follow-up (t1 vs. t2: *p =* 0.002).

In the ACT + FB group the change over time was significant in self-efficacy related to the adoption of exercise (*χ*^2^ = 12.310, *p =* 0*.*002), showing significant change between baseline and three-month follow-up (t1 vs. t2: *p* = 0.021). The change in self-efficacy when facing the barriers of exercise was significant (*χ*^2^ = 17.388, *p <* 0.001) between baseline and three- month follow-up (t1 vs. t2: *p* = 0.001), as well as, the change of action (*χ*^2^ = 46.562, *p <* 0*.*001; t1 vs. t2: *p <* 0.001) and coping planning related to own exercise intentions (*χ*^2^ = 96.073, *p <* 0.001; t1 vs. t2: *p <* 0.001). In addition, the change over time was significant in the acceptance of psychological and physical discomfort related to physical activity exercise intentions (*χ*^2^ = 38.499, *p <* .001; t1 vs. t2: *p <* 0.001).

The results showed statistically significant difference in change over time between the groups, favouring the ACT + FB group in the self-efficacy related to the adoption of exercise (*χ*^2^ = 7.212, *p =* 0.027; t1 vs. t2: *p* = 0.023) and self-efficacy when facing the barriers of exercise (*χ*^2^ = 11.600, *p =* 0.003; t1 vs. t2: *p* = 0.002). In addition, the greater improvements were also observed over time in the ACT + FB group in the change of action (*χ*^2^ = 15.824, *p <* 0.001;t1 vs. t2: *p* < 0.001) and coping planning related to own exercise intentions (*χ*^2^ = 24.210, *p <* 0.001; t1 vs. t2: *p* < 0.001), as well as in the acceptance of psychological and physical discomfort related to physical activity (*χ*^2^ = 15.621, *p <* 0.001.; t1 vs. t2: *p =* 0.009) compared to FB group.

### Change in the rank order stability

#### Physical activity behaviour

**Figure 2:**
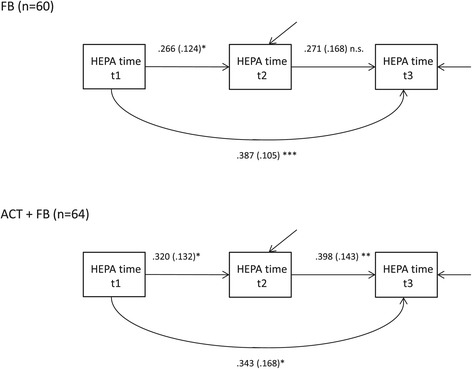
**The path model of time spent on health-enhancing physical activity (HEPA).** The path model was fitted in FB and ACT+FB groups. Standardized parameter estimates and standard errors (s.e.) are presented. n.s. p≥0.05;*p<0.05; **p<0.01; ***p<0.001.

The path models were fitted for three physical activity measures, HEPA time (see Figure 2), MVPA time, and self-reported physical activity (see Additional file 2: Figure S2 and Additional file 3: Figure S3). Each of the freely estimated path models showed a higher rank order stability for the ACT + FB group between t2 and t3 than for the FB group. However, the constrained model with equal regression coefficients across groups was accepted for HEPA time (*χ*^2^(3) = 1.13, *p* = 0.77), MVPA time (*χ*^2^(3) = 3.21, *p* = 0.36), and self-reported physical activity (*χ*^2^(3) = 7.03, *p* = 0.07), which showed that there was no statistical difference in the individual stability between the groups.

### Re-analysis among the participants without depressive symptoms (BDI-II <14) in the physical activity behaviour

Based on the earlier research results about the negative association of depression and physical activity [2-6] and because we observed almost statistical difference in the prevalence for at least mild depression (BDI-II ≥ 14, *p* = 0.056) between the FB and ACT + FB groups (see Table 2), the intervention effects were also analysed among the participants, who had none at all or a minimal amount of depressive symptoms (BDI < 14). Thereby 23 participants (BMI-II ≥ 14) were excluded from the analysis.

First, we evaluated the effects and change in the mean levels of physical activity in this subgroup. No difference was observed between the FB and ACT + FB groups in the change of HEPA time (*χ*^2^ = 3.09, *p* = 0.213), MVPA time (*χ*^2^ = 2.535, *p* = 0.281), or self-reported physical activity (*χ*^2^ = 1.940, *p* = 0.379) over time.

**Figure 3:**
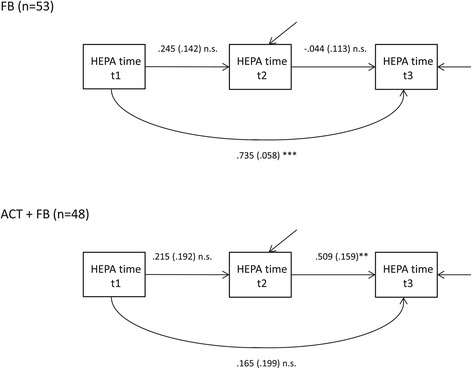
**The path model of time spent on health-enhancing physical activity (HEPA), among non-depressed participants (BDI-II<14).** The path model was fitted in FB and ACT+FB groups. Standardized parameter estimates and standard errors (s.e.) are presented. n.s. p≥0.05;*p<0.05; **p<0.01; ***p<0.001.

Second, we evaluated the rank order stability of physical activity among participants without depressive symptoms. The constrained model for HEPA time with equal regression coefficients across groups was rejected (*χ*^2^(3) = 9.14, *p* = 0.03) and the estimation results of the saturated model are presented in Figure 3. The test of equality of each regression coefficient showed a significant difference in regression coefficients between the FB and ACT + FB groups between t2 and t3 (*b* = −0.04 vs. *b =* 0.51, *p* = 0.001).

The constrained model for MVPA time with equal regression coefficients across groups was rejected (*χ*^2^(3) = 12.90, *p* = 0.005) and the estimation results of saturated model are presented in the Additional file 4: Figure S4. The test of equality of each regression coefficient showed a significant difference in regression coefficients between the FB and ACT + FB groups between t2 and t3 (*b* = 0.03 vs. *b* = 0.64, *p* < 0.001) and between t1 and t3 (*b* = 0.78 vs. *b* = −0.04, *p* = 0.001).

The constrained model for self-reported physical activity was accepted (*χ*^2^(3) = 5.97, *p* = 0.11), showing no significant differences in regression coefficients between the FB and ACT + FB groups. The estimation results of saturated model are presented in Additional file 5: Figure S5.

To conclude our results among non-depressive participants, the rank order stability of physical activity was higher in the ACT + FB group compared to the FB group between the three-month and six-month follow-ups in objectively measured MVPA and HEPA time, which showed that participants in the ACT + FB group were able to maintain the improvement in their individual physical activity level after intervention more consistently than participants in FB group. In addition, the stability between the baseline and the six-month follow-up for the MVPA time was significantly higher in the FB group compared to the ACT + FB group, indicating the return to the baseline physical activity level. Unlike those in the ACT + FB group, most of the participants in the FB group had a high risk to regress back to the baseline. However, there was no difference between the groups in the individual stability for the self-reported physical activity.

## Discussion

As far as we know this randomized controlled trial assessed for the first time the effectiveness of the feedback only (FB) versus the combination of acceptance- and commitment-based group intervention, including self-monitoring of physical activity and feedback (ACT + FB) on physical activity and the cognitions related to exercise and physical activity among physically inactive adults. The further aim was to explore the stability of individual changes in physical activity level between the groups. Unlike the previous findings of using ACT to enhance physical activity [29,32], the results did not show the difference between the groups in the change of mean physical activity level over time. Similarly, positive changes in physical activity, but not significant between the groups, were observed also in a study of Katterman et al. [31] among young female adults in the weight prevention intervention. However, differences over time between the ACT + FB and FB groups were observed between the groups in the change of cognitions related to exercise and physical activity. The ACT + FB group improved more than FB group in adoption self-efficacy, self-efficacy when facing the barriers of exercise, the ability to plan and cope with their exercise routines as well as in the acceptance of psychological and physical discomfort related to physical activity over the time from baseline to three months follow-up . In addition, the re-analysis of rank-order stability of objectively measured physical activity among the non-depressive participants, showed that the changes in physical activity were better maintained at the individual level in the ACT + FB group compared to the FB group.

Based on the objective physical activity measures, mean HEPA time increased in both groups at the three- and six-month follow-ups. In practice, members of the ACT + FB group increased their weekly HEPA time by 39 min per week, from 44.8 at the baseline to 83.3 min per week at the six-month follow-up. Thus, they almost doubled their weekly HEPA time. In the FB group, the increase in HEPA time was 31 minutes per week (from 40.6 to 72.1 min per week) at the six-month follow-up. Even though there is no clear evidence for a dose–response relation between physical activity and health status, it appears that even small improvements in the physical activity level are beneficial. In earlier studies, the greatest improvements in health status were seen when previously sedentary people were able to increase their physical activity [14]. Regardless of the changes in the mean physical activity level, we observed large individual differences in the change of physical activity scores between different measurement points. It is known that behaviour change at the individual level rarely follows the average trajectories of change [58]. Thus, the rank order stability comprehends the interpretation of the results beside the changes in the mean levels, giving more individual perspective to the change, and it is especially encouraging when individual variance is high in the population [59]. In the present study, the results actually showed a different kind of development in the objectively measured physical activity between the FB and ACT + FB groups, among the individuals without depressive symptoms. Both the FB and ACT + FB groups increased their objectively measured HEPA time at the group level but, after the stability model estimation, a re- analysis of the data among the non-depressive participants revealed that the ACT + FB group showed significantly better stability between the three- and six-month follow-ups compared to the FB group. Based on the stability analyses in the FB group, the positive change in the objectively measured physical activity from the baseline to three months was not sustained at the individual level at the six-month follow-up. This refers to the fact that the maintenance of the mean level change was due to different persons’ improvement at the three- and at six-month follow-ups. In addition, the participants in the FB group had a high risk of regressing back to the baseline in their physical activity level at the six-month follow-up. The ACT + FB group stability at the individual level was significantly better than in the FB group, indicating that the acquired change after the intervention was also better maintained at the individual level at the six month follow-up among the non–depressive participants. A similar, but not significant, phenomenon was observed in the self-reported physical activity.

The results provided the evidence that the acceptance- and value-based intervention was notably effective in enhancing exercise-related cognitions, which may be easier than changing habitual behaviour such as physical activity [8]. The adoption self-efficacy and self-efficacy related to the barriers improved significantly in the ACT + FB group, as well as the ability to make action plans and to cope with difficulties related to exercising. These factors are important in the adoption and maintenance of a physically active lifestyle [58]. Also, the results related to the acceptance of psychological and physical discomfort related to physical activity are congruent with the underlying theory and methods of ACT, which emphasizes the role of psychological flexibility in the behaviour change process. Physically inactive individuals may face different kind of discomfort related to physical activity, because of their greater body weight, worse physical fitness, and psychological uncertainty in physical activity situations. Thus, the acceptance of this discomfort related to physical activity by in**c**reasing psychological flexibility may be partly contributing to the successful behaviour change.

The results of this study suggest that the intervention in the acceptance and commitment based group did not lead to an increase of additional minutes of physical activity, compared to the feedback group from baseline to 3 and 6 month follow ups. The feedback only condition was also efficacious in improving mean physical activity. The benefits of the ACT based group sessions seem to be linked to a better maintenance of the changes, even very small changes, at the individual level, rather than an increase in exercise time or effectiveness.

Similar findings have been observed with a study of smoking cessation [37], which showed no differences between conditions at post treatment, but participants in the ACT condition had better long-term smoking outcomes at 1-year follow-up and these outcomes were mediated by the acceptance related skills. Based on earlier findings ACT intervention may be also beneficial in increasing high-intensity exercise tolerance time and post-exercise enjoyment, and reduce perceived effort in low-active women [60]. Thus, along the maintenance of regular physical activity behaviour, ACT related skills may be beneficial in tolerating the aversive effects of a single bout of exercise.

### Strengths of the study

This randomized control trial increases our knowledge of ACT-based methods that could be used to increase physical activity among physically inactive persons. This study provides a new model and different techniques (e.g., value clarification, mindfulness skills) to motivate physically inactive adults to change their lifestyle physically more active. Changes in physical activity level were measured objectively by accelerometers. Analyses were made with a sophisticated treatment of missing data by using both at mean level and rank order stability.

### Limitations of the study

The results can only be generalized for physically inactive women who voluntarily attend a value-based lifestyle program. Although objective physical activity measurements strengthen the study, this method also has its limitations. Many types of physical activities, such as cycling, swimming and gym training, are not registered by a waist-worn accelerometer. Another limitation is related to the effects of the physical activity measurement itself on the results. Wearing the accelerometer and receiving feedback for one’s physical activity during the seven-day measurement period may have had an effect on physical activity behaviour during the measurement period. This effect may have been even more pronounced in the feedback group, because the physical activity measurements and feedback after the measurement was the only action in which they were involved. Thus, giving feedback of the physical activity measurement only after the whole study period would have diminished this possible measurement effect. In addition, it must be acknowledged that there is a possibility that the benefits of ACT + FB interventions on cognitions might be the results of extra attention the participants received compared to the feedback condition. However, in contrast, based on the results we can conclude that receiving only feedback is not enough to modify the cognitions.

## Conclusion

This study showed that acceptance- and commitment-based group intervention, including self-monitoring of physical activity and feedback, may enhance the cognitions related to exercise and physical activity. In addition, the results of the re-analysis suggest that ACT based intervention may improve the stability of physical activity change, compared to a treatment which includes only feedback on the monitored physical activity among non-depressed participants. Providing only feedback on one’s physical activity level may increase physical activity among physically inactive adults at group level in the short term, but this improvement seems to be maintained at the individual level only temporarily. Future studies should focus more on mediators e.g. acceptance that are linked to the behaviour change in physical activity and explore the long-term maintenance of the changes in the ACT based interventions. Due to the high variation in the changes of physical activity level, future studies are encouraged to include individual level analysis as well. In addition, depressive symptoms are important to take into account when studying changes in physical activity behaviour among physically inactive adults.

## Additional files

Additional file 1: Figure S1. Individual trajectories of health enhancing physical activity (HEPA). The feedback group (FB above) and acceptance- and commitment-based group (ACT + FB group below). Additional file 2: Figure S2. The path model of time spent on moderate-to-vigorous intensity physical activity (MVPA). The path model was fitted in FB and ACT + FB groups. Standardized parameter estimates and standard errors (s.e.) of freely estimated model are presented. n.s. p≥0.05;*p<0.05; **p<0.01; ***p<0.001. Additional file 3: Figure S3. The path model of time spent on self-reported physical activity. The path model was fitted in FB and ACT+FB groups. Standardized parameter estimates and standard errors (s.e.) are presented. n.s. p≥0.05;*p<0.05; **p<0.01; ***p<0.001. Additional file 4: Figure S4. The path model of time spent on moderate-to-vigorous intensity physical activity (MVPA) among non-depressed participants (BDI-II<14). The path model was fitted in FB and ACT+FB groups. Standardized parameter estimates and standard errors (s.e.) are presented. n.s. p≥0.05;*p<0.05; **p<0.01; ***p<0.001. Additional file 5: Figure S5. The path model of time spent on self-reported physical activity among non-depressed participants (BDI-II<14). The path model was fitted in FB and ACT+FB groups. Standardized parameter estimates and standard errors (s.e.) are presented. n.s. p≥0.05;*p<0.05; **p<0.01; ***p<0.001.

## Abbreviations

ACT: Acceptance and Commitment Therapy
FB: Feedback
CPM: Counts per minute
MVPA: Moderate-to-vigorous intensity physical activity
HEPA: Health-enhancing physical activity
BDI-II: Beck depression inventory
PA-AAQ: Physical activity acceptance questionnaire
