# Supplementary material for: Towards a physically more active lifestyle based on one’s own values: the results of a randomized controlled trial among physically inactive adults
Source: BMC Public Health. 2015 Mar 18;15:260. doi: 10.1186/s12889-015-1604-x (PMC4371624; doi:10.1186/s12889-015-1604-x)
Supplement: Additional file 1: Figure S1. — Individual trajectories of health enhancing physical activity (HEPA). The feedback group (FB above) and acceptance- and commitment-based group (ACT + FB group below). [file 12889_2015_1604_MOESM1_ESM.pptx]

## Slide 1
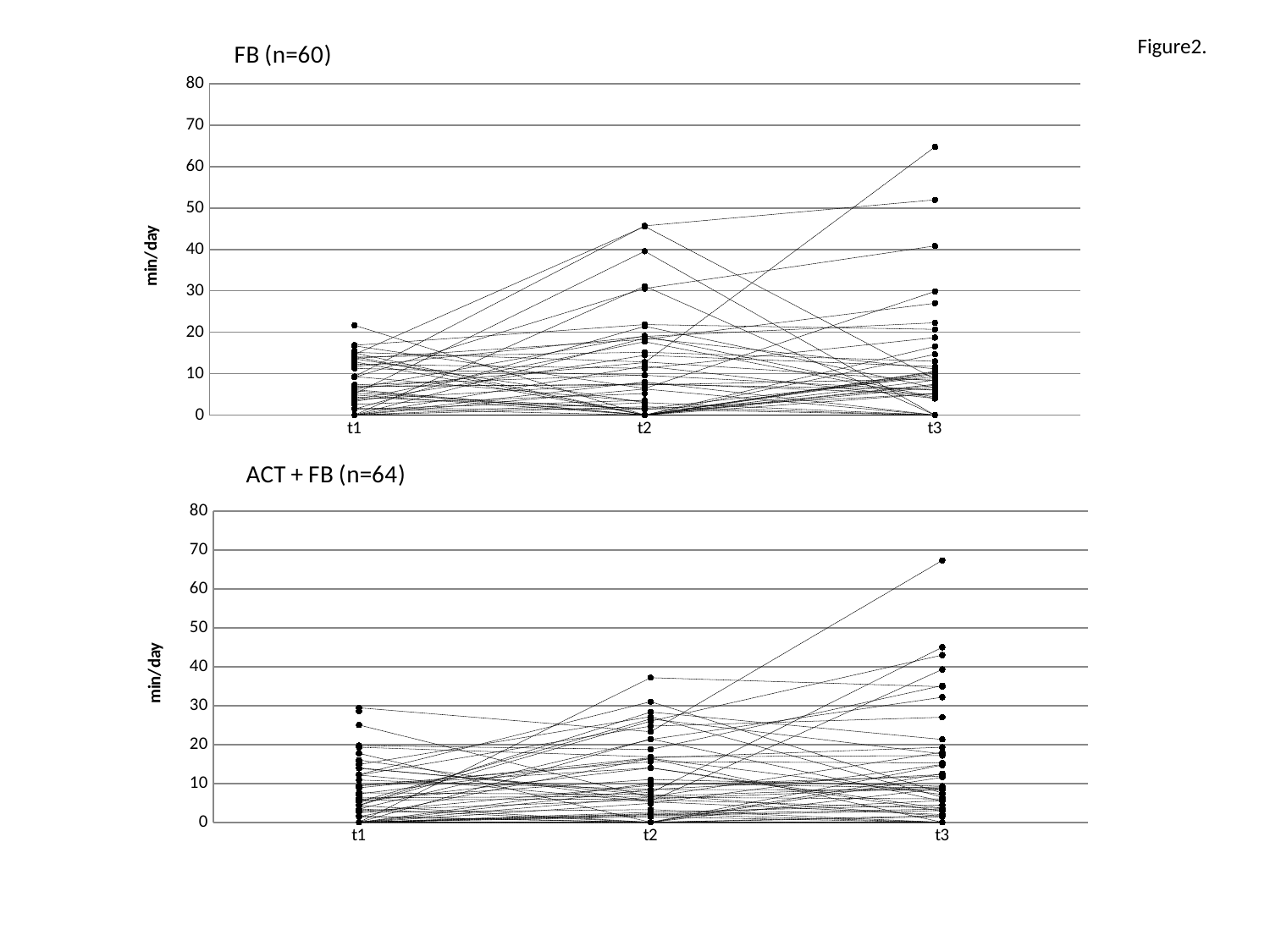

Figure2.
### Chart: FB (n=60)
| Category | | | | | | | | | | | | | | | | | | | | | | | | | | | | | | | | | | | | | | | | | | | | | | | | | | | | | | | | | | | | |
|---|---|---|---|---|---|---|---|---|---|---|---|---|---|---|---|---|---|---|---|---|---|---|---|---|---|---|---|---|---|---|---|---|---|---|---|---|---|---|---|---|---|---|---|---|---|---|---|---|---|---|---|---|---|---|---|---|---|---|---|---|
| t1 | 0.0 | 11.7143 | 5.2857 | 13.6667 | 11.2857 | 0.0 | 12.2857 | 14.0 | 12.8571 | 0.0 | 16.8571 | 0.0 | 13.5714 | 15.0 | 0.0 | 14.1429 | 14.6667 | 15.1429 | 0.0 | 0.0 | 5.1429 | 0.0 | 0.0 | 0.0 | 0.0 | 7.0 | 21.6667 | 6.8571 | 3.7143 | 5.8571 | 3.7143 | 0.0 | 4.3333 | 16.7143 | 3.2857 | 0.0 | 15.4286 | 9.2857 | 1.4286 | 0.0 | 7.2857 | 1.4286 | 1.5714 | 0.0 | 6.4286 | 0.0 | 0.0 | 2.5714 | 0.0 | 0.0 | 13.8 | 1.5714 | 0.0 | 9.2857 | 0.0 | 6.4286 | 6.1429 | 9.1429 | 0.0 | 4.8571 |
| t2 | 11.8333 | 19.0 | 1.4286 | 18.2857 | 9.6 | 0.0 | 11.2857 | 15.1667 | 0.0 | 0.0 | 21.8571 | 0.0 | 3.0 | 0.0 | 0.0 | 0.0 | 45.5714 | 12.8333 | 0.0 | 0.0 | 39.5714 | 8.0 | 6.2857 | 18.6 | 2.0 | 7.2857 | 0.0 | 7.4286 | 17.7143 | 0.0 | 7.7143 | 0.0 | 1.5714 | 6.4286 | 14.4286 | 31.1429 | None | 45.7143 | 21.4286 | None | 9.7143 | 3.5714 | 0.0 | 0.0 | 19.1429 | 0.0 | None | 5.2 | None | 3.0 | None | 1.4286 | 0.0 | 3.2857 | 0.0 | 12.6667 | 0.0 | 30.5714 | 0.0 | 0.0 |
| t3 | 4.0 | 22.2857 | 5.1429 | 27.0 | 6.0 | 7.2 | 18.7143 | 13.0 | 10.4286 | 8.4286 | 20.7143 | 0.0 | None | 10.5714 | 6.7143 | 16.5714 | 8.7143 | 8.2857 | 0.0 | 5.1667 | 0.0 | 0.0 | 0.0 | 11.1429 | 0.0 | 8.7143 | 14.6667 | 6.1429 | 4.0 | None | 5.0 | 0.0 | 9.2857 | 29.8571 | 11.7143 | 0.0 | None | 52.0 | 6.5714 | None | None | None | None | 7.4286 | 4.2857 | 0.0 | None | None | None | 0.0 | None | 0.0 | 0.0 | None | 0.0 | 64.8 | 10.1429 | 40.8571 | 9.8571 | 0.0 |
### Chart: ACT + FB (n=64)
| Category | | | | | | | | | | | | | | | | | | | | | | | | | | | | | | | | | | | | | | | | | | | | | | | | | | | | | | | | | | | | | | | | |
|---|---|---|---|---|---|---|---|---|---|---|---|---|---|---|---|---|---|---|---|---|---|---|---|---|---|---|---|---|---|---|---|---|---|---|---|---|---|---|---|---|---|---|---|---|---|---|---|---|---|---|---|---|---|---|---|---|---|---|---|---|---|---|---|---|
| t1 | 7.5714 | 0.0 | 1.5714 | 16.0 | 0.0 | 0.0 | 5.4286 | 6.2857 | 29.4286 | 12.2857 | 0.0 | 7.1429 | 5.2857 | 8.8571 | 12.1429 | 14.8571 | 0.0 | 0.0 | 0.0 | 4.3333 | 1.4286 | 6.2857 | 0.0 | 0.0 | 7.2857 | 0.0 | 3.4286 | 0.0 | 4.4286 | 2.5714 | 28.5714 | 0.0 | 0.0 | 1.4286 | 9.4286 | 0.0 | 0.0 | 7.0 | 14.0 | 0.0 | 15.7143 | 0.0 | 0.0 | 0.0 | 5.2857 | 0.0 | 3.2857 | 25.0 | 0.0 | 19.2857 | 17.7143 | 15.0 | 12.1429 | 19.7143 | 13.8571 | 4.2857 | 10.8571 | 5.8571 | 3.0 | 5.7143 | 9.4 | 3.4286 | 0.0 | 2.5714 |
| t2 | 7.1429 | 2.0 | 0.0 | 5.4286 | 0.0 | 0.0 | None | 6.6667 | 23.2857 | 31.0 | None | None | 26.5714 | 16.5714 | 5.2857 | 27.1429 | 6.5 | 8.4286 | 0.0 | 26.0 | 16.4286 | None | 37.1667 | 0.0 | 16.4 | 1.4286 | 21.2857 | 2.5714 | 28.3333 | 3.25 | None | None | 10.0 | 0.0 | 15.5714 | 0.0 | 0.0 | None | 7.0 | 1.7143 | None | 2.0 | None | 4.8571 | 14.0 | 2.2 | None | 6.4286 | None | 16.8333 | 0.0 | None | 24.7143 | 18.7143 | 7.5714 | 0.0 | 8.4286 | 11.0 | 5.8333 | 6.0 | 14.0 | 0.0 | 21.4286 | 9.5 |
| t3 | 1.5714 | 5.6 | 1.7143 | 39.2857 | 1.4286 | 0.0 | None | 9.1429 | 67.3333 | 7.5 | 5.6667 | None | 17.5714 | 19.2857 | 2.0 | 6.4286 | 18.0 | 8.2857 | 11.5714 | 43.0 | 7.1429 | None | 34.8571 | 8.7143 | 0.0 | 0.0 | 32.1429 | 4.4286 | 21.2857 | 0.0 | None | None | 8.8 | 0.0 | 15.2857 | 12.5 | 0.0 | None | None | 3.0 | 5.4286 | 3.4 | 0.0 | 14.8571 | 3.5714 | 0.0 | None | 6.0 | None | 17.1429 | 0.0 | None | 27.0 | 35.1429 | 45.0 | 14.7143 | 12.2857 | 9.2857 | 3.5714 | 8.8571 | 2.8571 | 0.0 | 5.4286 | 11.8571 |
