# Supplementary figures and images for: Towards a physically more active lifestyle based on one’s own values: the results of a randomized controlled trial among physically inactive adults
Source: BMC Public Health. 2015 Mar 18;15:260. doi: 10.1186/s12889-015-1604-x (PMC4371624; doi:10.1186/s12889-015-1604-x)

## Slide 1
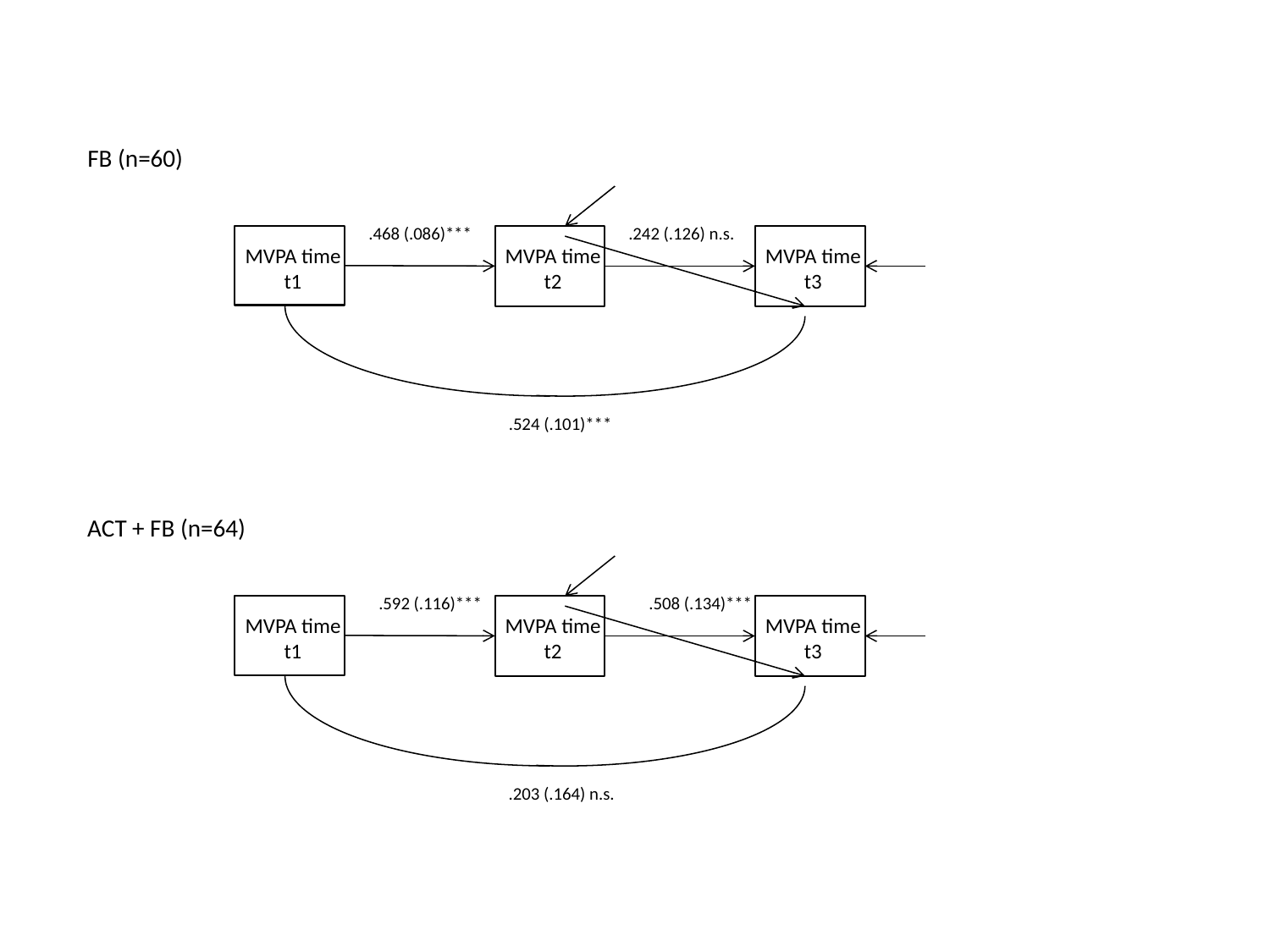

FB (n=60)
.468 (.086)***
.242 (.126) n.s.
MVPA time
t1
MVPA time
t2
MVPA time
t3
.524 (.101)***
ACT + FB (n=64)
.592 (.116)***
.508 (.134)***
MVPA time
t1
MVPA time
t2
MVPA time
t3
.203 (.164) n.s.

Supplement: Additional file 2: Figure S2. — The path model of time spent on moderate-to-vigorous intensity physical activity (MVPA). The path model was fitted in FB and ACT + FB groups. Standardized parameter estimates and standard errors (s.e.) of freely estimated model are presented. n.s. p≥0.05;*p<0.05; **p<0.01; ***p<0.001. [file 12889_2015_1604_MOESM2_ESM.pptx]

## Slide 1
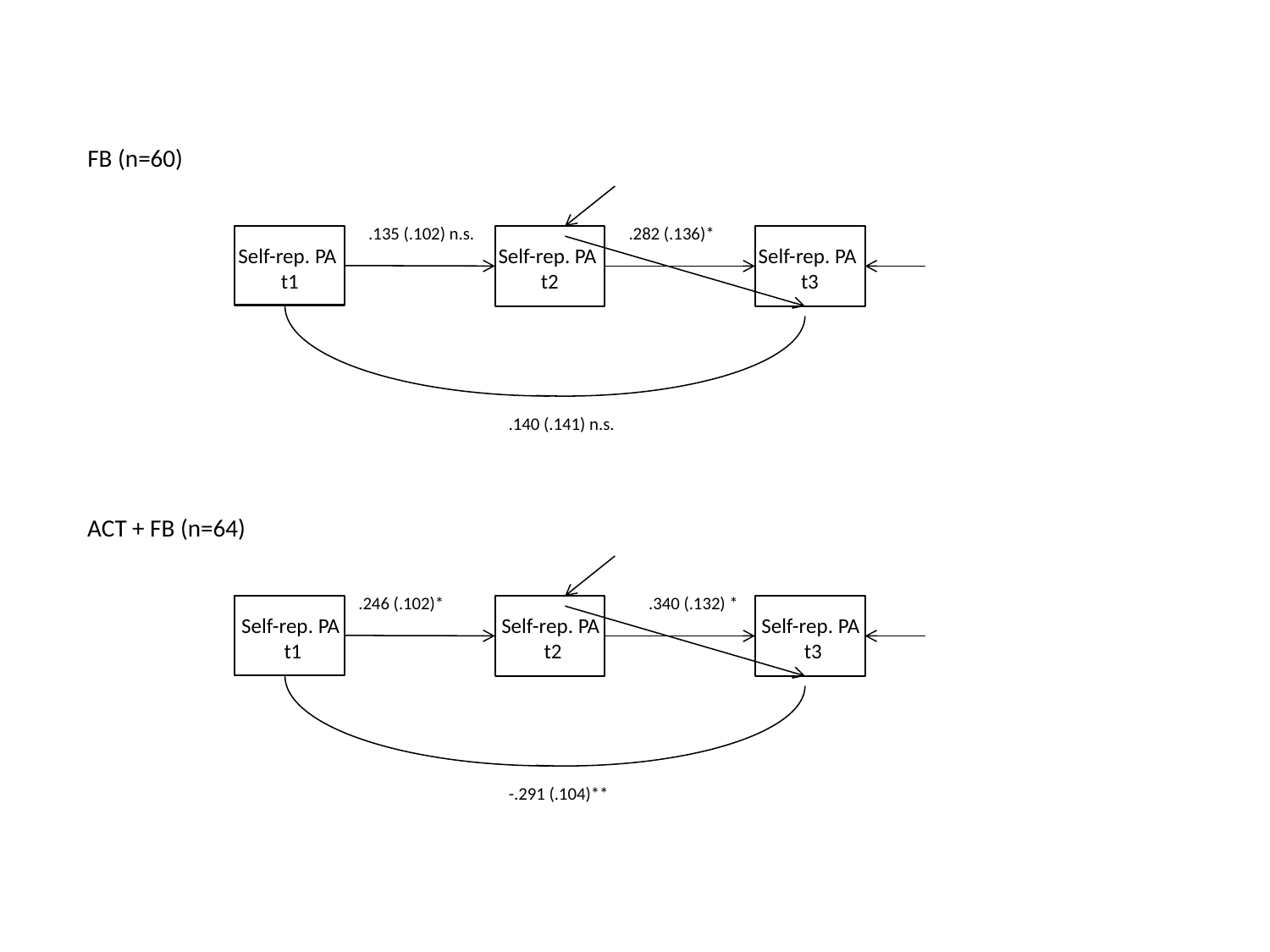

FB (n=60)
.135 (.102) n.s.
.282 (.136)*
Self-rep. PA
t1
.140 (.141) n.s.
Self-rep. PA
t2
Self-rep. PA
t3
ACT + FB (n=64)
.246 (.102)*
.340 (.132) *
Self-rep. PA
t1
Self-rep. PA
t2
Self-rep. PA
t3
-.291 (.104)**

Supplement: Additional file 3: Figure S3. — The path model of time spent on self-reported physical activity. The path model was fitted in FB and ACT+FB groups. Standardized parameter estimates and standard errors (s.e.) are presented. n.s. p≥0.05;*p<0.05; **p<0.01; ***p<0.001. [file 12889_2015_1604_MOESM3_ESM.pptx]

## Slide 1
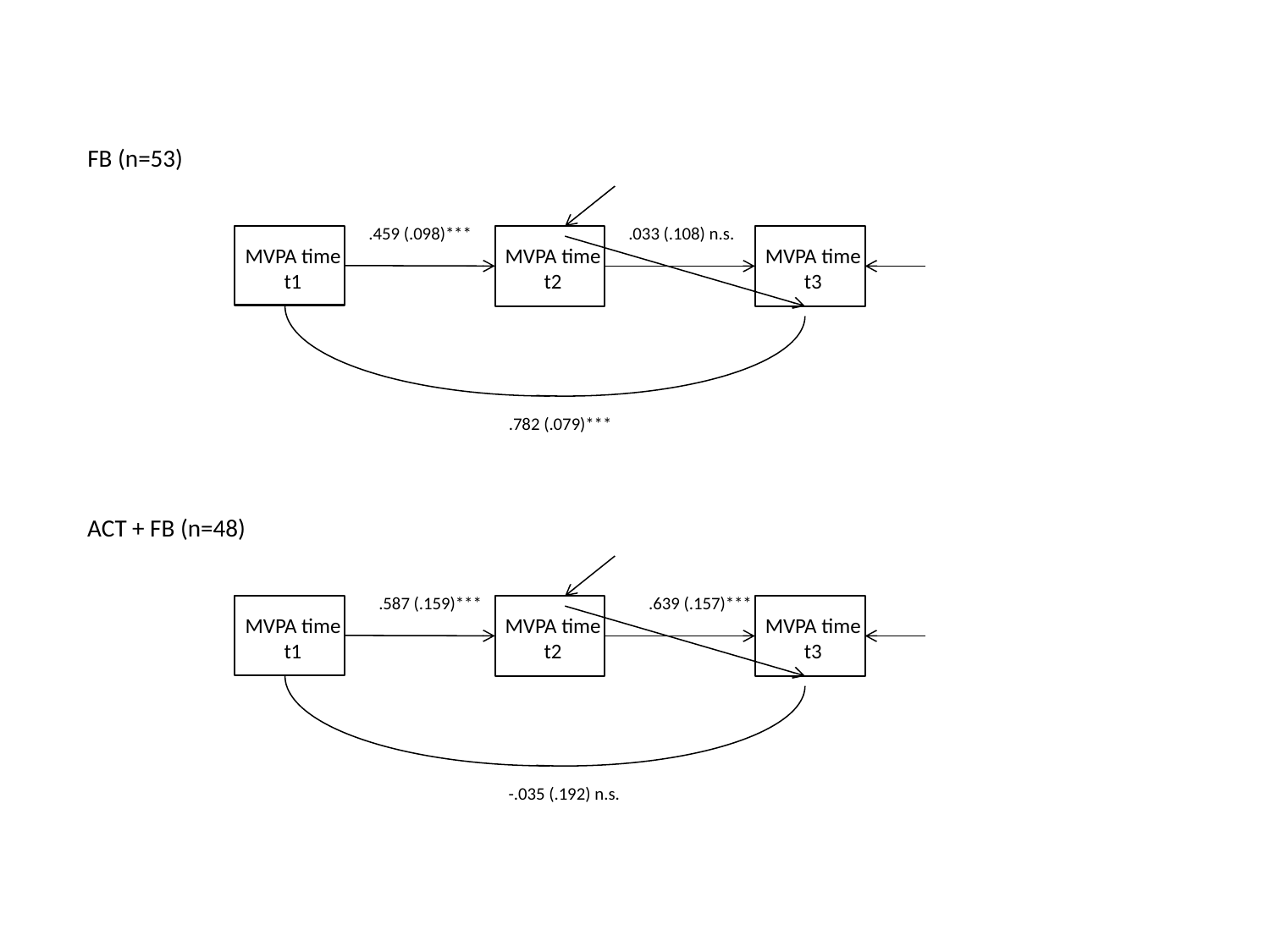

FB (n=53)
.459 (.098)***
.033 (.108) n.s.
MVPA time
t1
MVPA time
t2
MVPA time
t3
.782 (.079)***
ACT + FB (n=48)
.587 (.159)***
.639 (.157)***
MVPA time
t1
MVPA time
t2
MVPA time
t3
-.035 (.192) n.s.

Supplement: Additional file 4: Figure S4. — The path model of time spent on moderate-to-vigorous intensity physical activity (MVPA) among non-depressed participants (BDI-II<14). The path model was fitted in FB and ACT+FB groups. Standardized parameter estimates and standard errors (s.e.) are presented. n.s. p≥0.05;*p<0.05; **p<0.01; ***p<0.001. [file 12889_2015_1604_MOESM4_ESM.pptx]
