# Supplementary material for: Towards a physically more active lifestyle based on one’s own values: the results of a randomized controlled trial among physically inactive adults
Source: BMC Public Health. 2015 Mar 18;15:260. doi: 10.1186/s12889-015-1604-x (PMC4371624; doi:10.1186/s12889-015-1604-x)
Supplement: Additional file 5: Figure S5. — The path model of time spent on self-reported physical activity among non-depressed participants (BDI-II<14). The path model was fitted in FB and ACT+FB groups. Standardized parameter estimates and standard errors (s.e.) are presented. n.s. p≥0.05;*p<0.05; **p<0.01; ***p<0.001. [file 12889_2015_1604_MOESM5_ESM.pptx]

## Slide 1
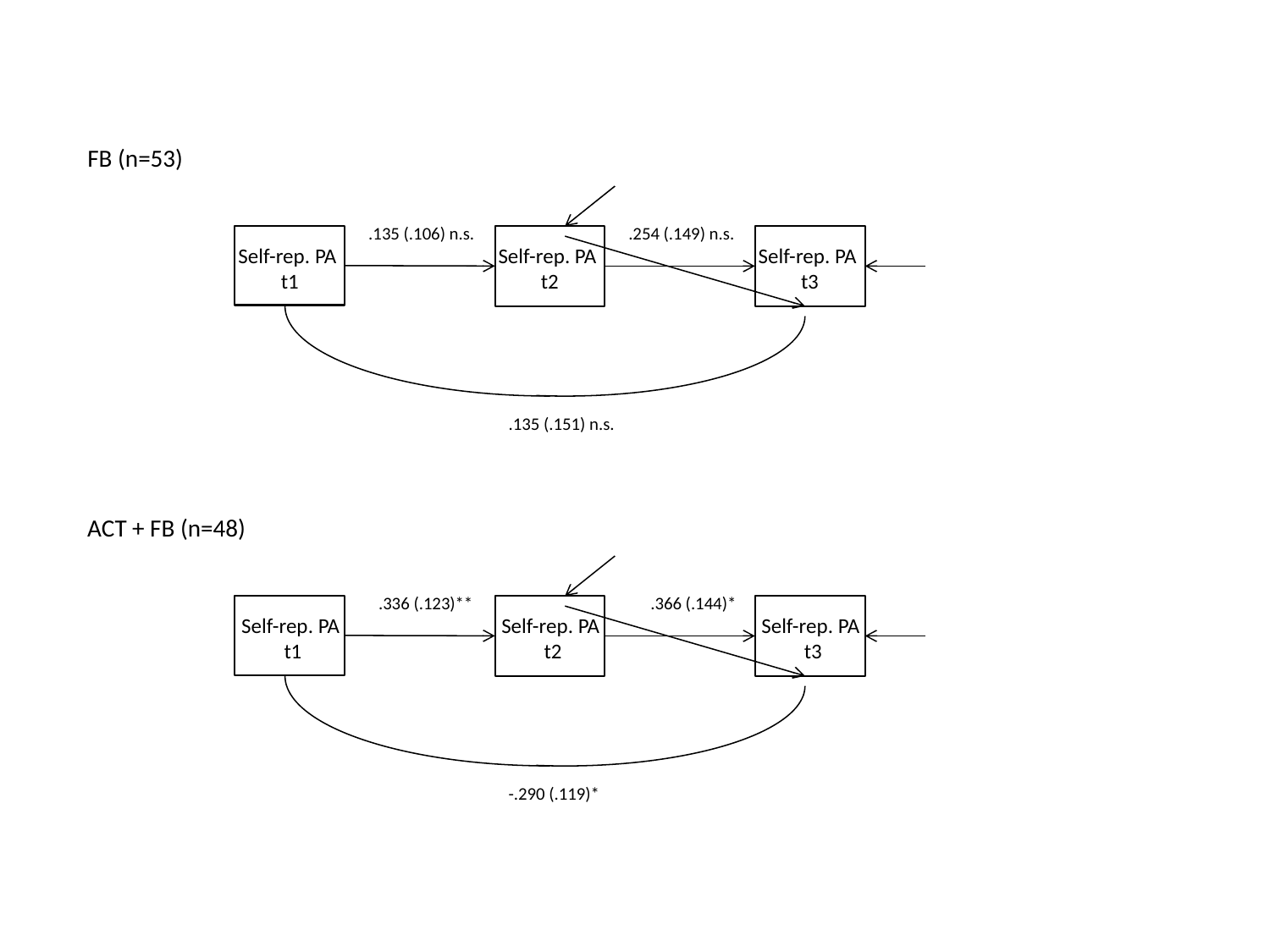

FB (n=53)
.135 (.106) n.s.
.254 (.149) n.s.
Self-rep. PA
t1
.135 (.151) n.s.
Self-rep. PA
t2
Self-rep. PA
t3
ACT + FB (n=48)
.336 (.123)**
.366 (.144)*
Self-rep. PA
t1
Self-rep. PA
t2
Self-rep. PA
t3
-.290 (.119)*
